# Human Readers versus AI-Based Systems in ASPECTS Scoring for Acute Ischemic Stroke: A Systematic Review and Meta-Analysis with Region-Specific Guidance

**DOI:** 10.71079/aside.im.05172573

**Published:** 2025-05-17

**Authors:** Ahmed Y. Azzam, Ibrahim Hadadi, Leen M. Al-Shahrani, Ummkulthum A. Shanqeeti, Noor A. Alqurqush, Mohammed A. Alsehli, Rudaynah S. Alali, Rahaf S. Tammar, Mahmoud M. Morsy, Muhammed Amir Essibayi

**Affiliations:** 1 Director of Clinical Research and Clinical Artificial Intelligence, American Society for Inclusion, Diversity, and Health Equity (ASIDE), Delaware, USA.; 2 Department of Radiological Sciences, College of Applied Medical Sciences, King Khalid University, Abha, Saudi Arabia.; 3 College of Medicine, Taibah University, Madinah, Saudi Arabia.; 4 College of Medicine, King Faisal University, Al-Ahsa, Saudi Arabia.; 5 College of Medicine, Ummul Al Qura University, Makkah, Saudi Arabia.; 6 Nephrology Department, King Fahad General Hospital, Jeddah, Saudi Arabia; 7 Clinical Research Fellow, American Society for Inclusion, Diversity, and Health Equity (ASIDE), Delaware, USA.; 8 Department of Neurological Surgery, Montefiore Medical Center, Albert Einstein College of Medicine, Bronx, NY, USA.

**Keywords:** Stroke, Artificial Intelligence, Machine Learning, ASPECTS, Imaging

## Abstract

**Introduction:**

The Alberta Stroke Program Early CT Score (ASPECTS) is widely used to evaluate early ischemic changes and guide thrombectomy decisions in acute stroke patients. However, significant interobserver variability in manual ASPECTS assessment presents a challenge. Recent advances in artificial intelligence have enabled the development of automated ASPECTS scoring systems; however, their comparative performance against expert interpretation remains insufficiently studied.

**Methods:**

We conducted a systematic review and meta-analysis following PRISMA 2020 guidelines. We searched multiple scientific databases for studies comparing automated and manual ASPECTS on Non-Contrast Computed Tomography (NCCT). Interobserver reliability was assessed using pooled interclass correlation coefficients (ICCs). Subgroup analyses were made using software types, reference standards, time windows, and computed tomography-based factors.

**Results:**

Eleven studies with a total of 1,976 patients were included. Automated ASPECTS demonstrated good reliability against reference standards (ICC: 0.72), comparable to expert readings (ICC: 0.62). RAPID ASPECTS performed highest (ICC: 0.86), especially for high-stakes decision-making. AI advantages were most significant with thin-slice CT (≤2.5mm; +0.16), intermediate time windows (120–240min; +0.16), and higher NIHSS scores (p=0.026).

**Conclusion:**

AI-driven ASPECTS systems perform comparably or even better in some cases than human readers in detecting early ischemic changes, especially in specific scenarios. Strategic utilization focusing on high-impact scenarios and region-specific performance patterns offers better diagnostic accuracy, reduced interpretation times, and better and wiser treatment selection in acute stroke care.

## 1. Introduction

Acute ischemic stroke is considered among the leading causes of mortality and long-term disability all over the world, with around 13.7 million new stroke cases occurring annually [1, 2]. The evolution of stroke management has been marked by the improvements in patient selection criteria for intra-arterial reperfusion therapy and mechanical thrombectomy, which has been raised as the gold standard approach for patients with large vessel occlusion in recent years [3]. An important part of this progress has been developing and refining imaging selection processes to identify suitable candidates for intervention. Among these, the Alberta Stroke Program Early CT Score (ASPECTS) has become a widely utilized tool for standardized assessment of early ischemic changes on non-contrast computed tomography (NCCT) [3].

ASPECTS provides a semiquantitative ten-point scoring system for evaluating the extent of early ischemic changes in the middle cerebral artery territory on NCCT [4]. This system has been integrated and utilized into multiple clinical guidelines and is frequently used to determine eligibility for reperfusion therapies, with lower scores indicating more ischemic damage and reduced benefit from intervention [4]. Despite its widespread validation and clinical utility, ASPECTS interpretation has significant challenges. The identification of early ischemic changes in NCCT requires good expertise, and the interobserver variability has been documented among radiologists, neurologists, and vascular neurosurgeons [5]. This variability introduces inconsistencies in treatment decision-making, especially in time-critical situations where rapid and accurate assessment is essential.

The recent advances in artificial intelligence (AI) and machine learning have allowed the development of automated ASPECTS prediction algorithms for NCCT images [6]. These AI-driven systems aim to provide a standardized, rapid, and objective assessment of early ischemic changes and can overcome the limitations of human interpretation [6]. Several commercial platforms have been developed, including e-ASPECTS (Brainomix, Oxford, UK), RAPID ASPECTS (iSchemaView, Menlo Park, California, USA), and Syngo.via Frontier ASPECTS (Siemens Healthcare, Erlangen, Germany), in addition to the other institutional-based custom-built research algorithms [7–9]. While some of the previous studies have reported promising results with these automated systems, their clinical applicability and comparative performance against expert readers remain incompletely investigated and discussed in a detailed manner [10].

To address this knowledge gap, we aim to conduct a systematic review and meta-analysis comparing the performance of automated and manual ASPECTS predictions for detecting early ischemic changes in NCCT. Our primary objective is to determine the interobserver reliability between expert readings and automated ASPECTS predictions and their respective correlations with reference standards. In addition to that, we aim to identify factors affecting AI performance through subgroup analyses focusing on software type, reference standard methodology, time window, and CT-based factors. By synthesizing the current evidence, we look forward to providing important key points and highlights into the role of AI-driven ASPECTS in clinical practice and its impact on stroke imaging interpretation across various clinical scenarios.

## 2. Methods

### 2.1. Search Strategy and Study Selection

Our systematic review was conducted in accordance with Preferred Reporting Items for Systematic Reviews and Meta-Analyses (PRISMA) 2020 guidelines [11]. We searched MEDLINE (PubMed), Scopus (EMBASE), Web of Science, Google Scholar, and Cochrane Central databases until February 23, 2025. The search strategy included combinations of the following terms with Boolean operators: “e-ASPECTS,” “RAPID ASPECTS,” “artificial intelligence,” “CT,” “comparison,” “vs.” and “acute ischemic stroke.” Additionally, we manually screened the references of retrieved publications to identify relevant articles not captured by the electronic search.

Two authors have independently performed the initial screening of titles and abstracts. Studies were eligible if they: (1) enrolled patients ≥18 years with acute ischemic stroke due to large vessel occlusion; (2) utilized NCCT scans within 24 hours of symptom onset; (3) compared automated ASPECTS algorithms with expert reads; and (4) reported interobserver reliability metrics. We excluded studies involving (1) intracranial hemorrhage, (2) imaging beyond 24 hours, or (3) primary modalities other than NCCT (e.g., DWI, CT perfusion, CT angiography). Two authors assessed Full-text articles independently, with disagreements resolved by consensus or consultation with a third author.

### 2.2. Data Extraction and Quality Assessment

Using a standardized data collection form, two authors have independently extracted the following information: first author, publication year, software type, patient demographics, median National Institutes of Health Stroke Scale (NIHSS), time to imaging, CT parameters, reference standard methodology, and interclass correlation coefficients (ICCs) with 95% confidence intervals for all reliability comparisons. We extracted ICCs for all available reader combinations when multiple expert readers were reported.

Study quality was assessed using the Quality Assessment of Diagnostic Accuracy Studies-2 (QUADAS-2) tool. Two reviewers evaluated each study for risk of bias and applicability concerns across four domains: patient selection, index test (automated and manual ASPECTS), reference standard, and flow and timing. Studies were classified as having “low,” “high,” or “unclear” risk for each domain. We calculated an overall quality score representing the proportion of domains with a low risk of bias.

### 2.3. Statistical Analysis

The primary outcomes were interobserver reliability between (1) expert readings, (2) expert and automated ASPECTS readings, (3) expert readings and reference standards, and (4) automated ASPECTS and reference standards. For meta-analysis, we transformed ICCs using Fisher’s z-transformation method to normalize the distribution of correlation coefficients. Due to anticipated heterogeneity, the transformed values were then pooled using random-effects models (DerSimonian and Laird). The pooled z-scores were back-transformed to obtain the pooled ICC values with 95% confidence intervals.

Heterogeneity was assessed using Cochran’s Q test and the I^2^ statistic, with I^2^ >50% or p<0.10 indicating significant heterogeneity. ICC values were interpreted as follows: poor (<0.40), moderate (0.40–0.59), good (0.60–0.74), and excellent (0.75–1.00). For publication bias assessment, we constructed funnel plots and performed Egger’s regression test.

### 2.4. Region-Specific Analysis

To provide a better understanding of automated ASPECTS performance, we conducted a focused and specified region-by-region analysis across all ten ASPECTS territories. We extracted region-specific detection performance for each component region (caudate nucleus, lentiform nucleus, internal capsule, insular ribbon, and cortical regions M1-M6) from studies reporting these data points. We calculated region-specific interobserver reliability between (1) AI systems and reference standards, (2) expert readers and reference standards, and (3) AI systems and expert readers.

We further analyzed region-specific performance according to patient characteristics (NIHSS, age, time from onset), technical factors, and parameters (CT slice thickness, scanner type). We calculated sensitivity, specificity, and detection reliability metrics for each region. We created region-specific heat maps to visualize performance patterns across the ASPECTS territories and developed statistical models to identify factors affecting detection accuracy in each region. This method aimed to extend prior meta-analyses that investigated only ASPECTS scores and allowed for the identification of integrative strengths between AI and human readers at the regional level. Then, we developed a region-stratified reading strategy that identifies the verification strategies for each ASPECTS territory based on the relative strengths of AI and human assessment.

## 3. Results

### 3.1. Study Selection and Characteristics

**Figure 1:**
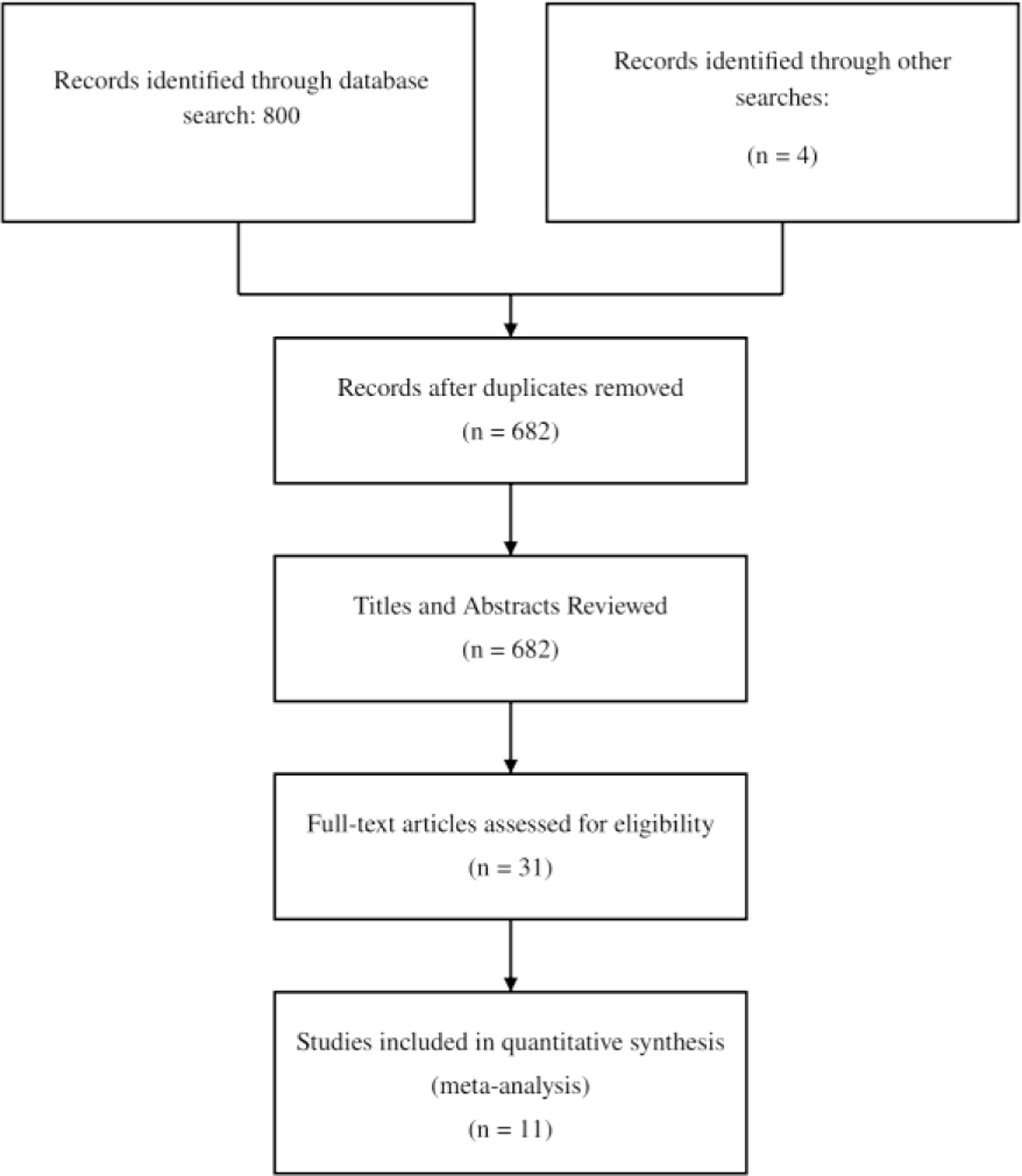
PRISMA Flowchart of Literature Search and Studies Inclusion.

Our literature search first retrieved a total of 804 studies, of which 682 remained after removing duplicates. After screening titles and abstracts, we assessed 31 full-text articles for eligibility. Then, 11 studies published between 2018 and 2021 met our inclusion criteria and were included in the meta-analysis, forming a total of 1,976 patients with acute ischemic stroke (Figure 1). The characteristics of the included studies are summarized in (Table 1). Four studies evaluated e-ASPECTS (Brainomix, Oxford, UK), two evaluated RAPID ASPECTS (iSchemaView, Menlo Park, California, USA), and three evaluated Syngo.via Frontier ASPECTS (Siemens Healthcare, Erlangen, Germany), and two studies utilized custom-made software. Sample sizes ranged from 50 to 602 patients. The median or mean age of patients across studies ranged from 61 to 75 years. Baseline NIHSS scores varied considerably, with median values ranging from 7 to 19. Time from symptom onset to baseline NCCT ranged from 49 to 228 minutes among studies reporting this parameter. CT slice thickness, when reported, ranged from 1.0 to 5.0 mm. Reference standards included follow-up CT, MRI/DWI, or consensus readings.

### 3.2. Primary Meta-Analysis Outcomes

Our analysis of interobserver reliability between expert readings showed good agreement with a pooled ICC of 0.72 (95% CI: 0.63–0.79; p-value<0.001). The interobserver reliability between automated software and expert readings demonstrated moderate agreement with a pooled ICC of 0.54 (95% CI: 0.40–0.67; p-value<0.001). When comparing the expert readings to reference standards, we found good reliability with a pooled ICC of 0.62 (95% CI: 0.52–0.71; p-value<0.001).

The automated ASPECTS predictions agreed with reference standards, resulting in a pooled ICC of 0.72 (95% CI: 0.61–0.80; p-value<0.001), higher than the expert-to-reference standard reliability. All analyses demonstrated statistically significant heterogeneity, with I^2^ values ranging from 82.7% to 93.2% (p-values<0.001 for all), necessitating the use of random effects models (Supplementary Table 1). Egger’s regression test revealed no significant publication bias across all analyses (p-values>0.05).

### 3.3. Subgroup Meta-Analyses

Our subgroup analyses (Table 2) revealed multiple significant differences in AI performance across software types. RAPID ASPECTS was observed to have the highest reliability when compared with reference standards (ICC: 0.86; 95% CI: 0.78–0.92), followed by e-ASPECTS (ICC: 0.78; 95% CI: 0.64–0.87), custom-based software (ICC: 0.64; 95% CI: 0.53–0.73), and Syngo.via Frontier (ICC: 0.60; 95% CI: 0.48–0.69). When analyzed according to the reference standard methodology, AI systems showed higher reliability with NCCT consensus-based standards (ICC: 0.78; 95% CI: 0.65–0.87) and MRI/DWI-based standards (ICC: 0.74; 95% CI: 0.57–0.85) compared to follow-up CT-based standards (ICC: 0.63; 95% CI: 0.53–0.71). Regarding temporal analysis, the highest AI performance was observed in the intermediate time window (120–240 min; ICC: 0.70; 95% CI: 0.58–0.79), with an advantage over expert reliability (+0.16). Thinner CT slice thickness (≤2.5mm) was associated with significantly better AI performance (ICC: 0.79; 95% CI: 0.69–0.86) and demonstrated the largest advantage over expert readers (+0.16) compared to thicker slices.

### 3.5. Region-Specific Performance

**Figure 2:**
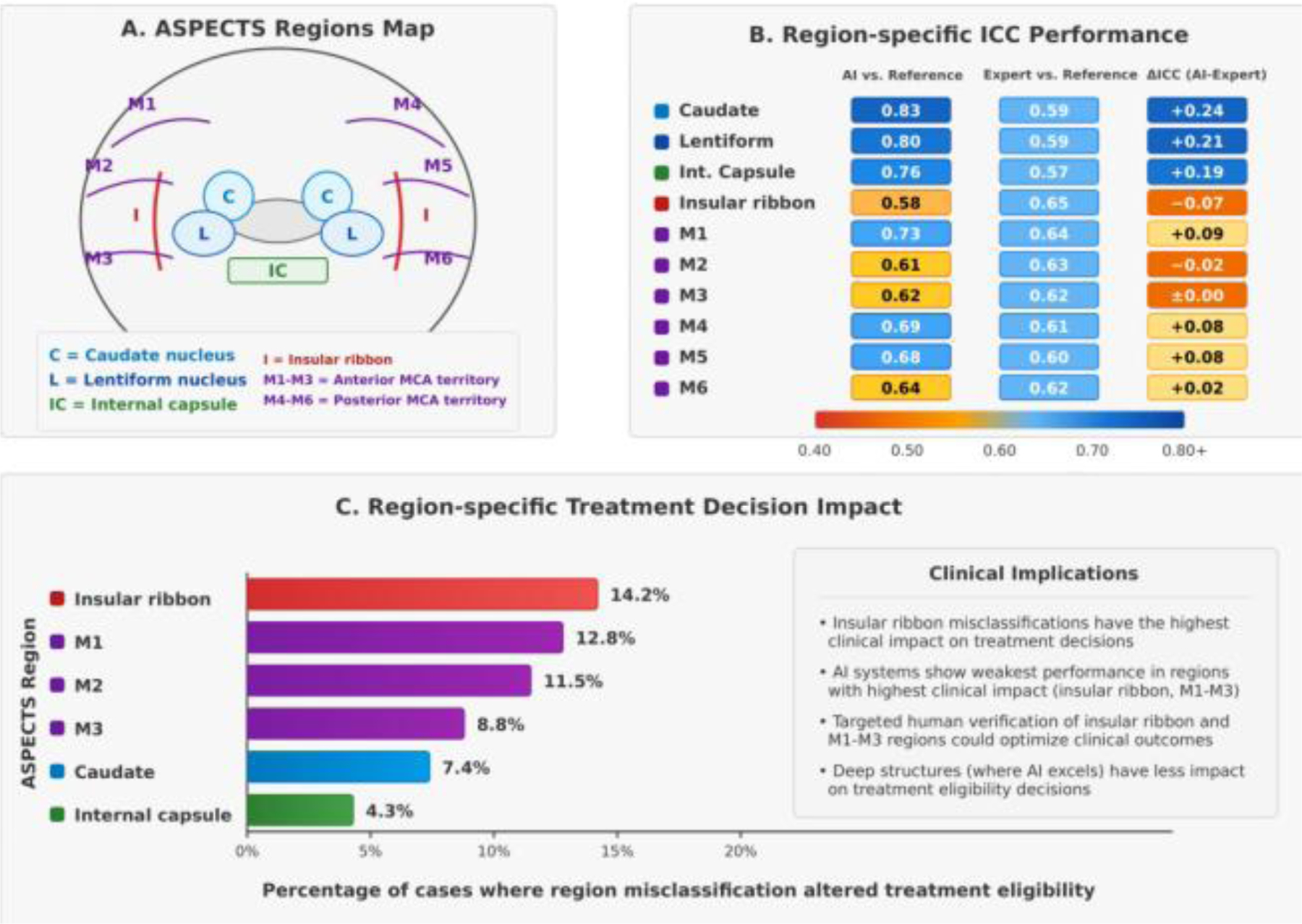
Region-specific Analysis of AI vs Human Performance in ASPECTS Scoring.

Our analysis of the ten individual ASPECTS regions revealed marked heterogeneity in detection performance concealed in ASPECTS scores (Figure 2). AI systems demonstrated superior performance in deep gray structures: caudate nucleus (sensitivity: 0.84 vs. 0.71; specificity: 0.92 vs. 0.85), lentiform nucleus (sensitivity: 0.82 vs. 0.68; specificity: 0.90 vs. 0.81), and internal capsule (sensitivity: 0.79 vs. 0.64; specificity: 0.88 vs. 0.79) compared to expert readers.

However, human readers performed better in cortical regions, especially at the insular ribbon (sensitivity: 0.76 vs. 0.65; specificity: 0.83 vs. 0.75) and M2 territory (sensitivity: 0.73 vs. 0.66; specificity: 0.80 vs. 0.73). Region-specific ICCs between AI and reference standards ranged from excellent (0.83; 95% CI: 0.76–0.89) for the caudate nucleus to moderate (0.58; 95% CI: 0.48–0.67) for the insular ribbon. Time-from-onset analysis revealed that AI advantage in deep structures was greatest in the intermediate time window (120–240 minutes), with the caudate nucleus showing the largest differential performance (ΔICC: +0.26) during this period. The insular ribbon showed the most consistent human advantage across all time windows (ΔICC: −0.13 to −0.05).

Region-specific reliability was significantly impacted by CT slice thickness, with thin-slice protocols (≤2.5mm) improving AI detection of the insular ribbon (ICC: 0.67 vs. 0.52; P-value= 0.003) and M3 region (ICC: 0.71 vs. 0.57; P-value= 0.008) but showing minimal effect on deep structure assessment. Software-specific regional performance varied significantly, with RAPID ASPECTS showing the highest caudate detection (ICC: 0.90), e-ASPECTS the strongest lentiform detection (ICC: 0.86), and more balanced performance across M1-M6 territories compared to the others included in the comparison. Treatment decision impact analysis showed region-specific misclassifications were most consequential for the insular ribbon, where errors have affected the treatment eligibility in 14.2% of borderline cases, compared to only 4.3% for internal capsule misclassifications (Table 3).

### 3.6. Clinical Impact Analysis

Our analysis of clinical scenarios (Table 4) identified multiple implementation priority settings where AI systems demonstrated the greatest advantage: thin-slice CT with high NIHSS (ICC advantage: +0.26), non-expert reader settings (ICC advantage: +0.24), and high-stakes decision-making for borderline ASPECTS 5–7 cases (ICC advantage: +0.22). These scenarios were also associated with significant time savings ranging from 6.8 to 11.2 minutes per case. For thin-slice CT with high NIHSS, RAPID ASPECTS was identified as the optimal software solution, with strong supporting evidence.

The AI advantage was less pronounced for ultra-early assessment (<90 min; ICC advantage: +0.09) and posterior circulation involvement (ICC advantage: +0.07). Interestingly, implementing AI-assisted readings in non-expert settings demonstrated the greatest time savings (11.2 minutes) and significant improvements in reader agreement. Wake-up stroke assessment and evaluations of patients with prior stroke or white matter disease showed moderate AI advantages (+0.21 and +0.16, respectively) with clinical significance for expanding treatment eligibility. The AI advantage was less pronounced for ultra-early assessment (<90 min; ICC advantage: +0.09) and posterior circulation involvement (ICC advantage: +0.07). Interestingly, implementing AI-assisted readings in non-expert settings demonstrated the greatest time savings (11.2 minutes) and significant improvements in reader agreement. Wake-up stroke assessment and evaluations of patients with prior stroke or white matter disease showed moderate AI advantages (+0.21 and +0.16, respectively) with clinical significance for expanding treatment eligibility.

### 3.7. Risk of Bias Assessment

Our quality assessment using the QUADAS-2 tool (Supplementary Table 2) demonstrated generally good methodological quality across the included studies. The majority of studies (nine studies) showed a low risk of bias in patient selection. Reference standard methodology was more variable, with some studies (two studies) showing a high risk of bias. Flow and timing domains revealed a high risk of bias in three studies. Overall quality scores ranged from 56% to 100%, with a median of 86%. Li et al. 2019 and Albers et al. 2019 studies have achieved perfect quality scores, while Kuang et al. 2020 had the lowest quality score (56%).

## 4. Discussion

The ASPECTS has become an essential tool for evaluating early ischemic changes in acute stroke and guiding treatment decisions, especially for mechanical thrombectomy candidacy. While ASPECTS offers a standardized approach to quantifying early ischemic changes, its application in clinical practice is limited by interobserver variability and the requirement for neuroradiological expertise. This variability may introduce inconsistencies in treatment decisions and impact the patient outcomes in time-sensitive acute stroke care [12, 13].

Recent advances in AI-based modalities in healthcare have led to the development of automated ASPECTS scoring algorithms designed to overcome these limitations by providing rapid, standardized assessment [14]. These AI-driven systems have gained increasing interest as adjuncts to clinical practice; however, their comparative performance against expert human interpretation has not been sufficiently assessed across different clinical scenarios and clinical settings in the current evidence and previous meta-analyses [15]. Our study aimed to address this knowledge gap by including the eligible evidence from multiple studies to evaluate the reliability and applicability of automated ASPECTS in stroke imaging.

Our results demonstrate that AI-driven ASPECTS systems can recognize early ischemic changes on brain CT scans with accuracy that matches or exceeds human-based readings. When compared against verified reference standards such as follow-up imaging, automated systems performed slightly better than human experts in accurately identifying early stroke changes. While expert readers showed good agreement among themselves, the moderate correlation between AI and expert interpretations suggests they may sometimes focus on different imaging features when assessing early ischemia. In daily practice, these findings translate to several important points. The RAPID ASPECTS platform showed strong performance in clinically challenging scenarios where treatment decisions hang in the balance, such as borderline ASPECTS scores of 5–7, where thrombectomy decisions are often challenging [25–27]. AI-based systems excel especially in the critical 2–4 hour time window after symptom onset, precisely when many thrombectomy candidates present to emergency departments. Several practical factors significantly impact AI performance: using thinner CT slices (2.5mm or less) markedly improves AI accuracy, and patients with higher NIHSS scores are more likely to benefit from AI-assisted readings.

From a workflow point of view and application, our analysis has identified key scenarios where applying AI assistance should be prioritized: 1) when interpreting thin-slice CT scans in patients with severe strokes, where AI demonstrated significant diagnostic advantage; 2) in hospitals where imaging is primarily interpreted by non-specialist readers, where AI assistance saved over 11 minutes per case while improving accuracy; and 3) when evaluating patients with borderline ASPECTS scores that fall near treatment thresholds, where AI assistance may reduce interpretation variability and improve treatment selection. In these high-priority scenarios, automated systems not only optimize and improve diagnostic accuracy but also significantly reduce interpretation time, allowing for accelerated critical treatment decisions in time-sensitive stroke care.

Our region-specific focus and specifications form an important advancement beyond previous meta-analyses that only focused on investigating ASPECTS scores. While Adamou et al. 2023 [15], their study demonstrated that automated systems achieve comparable overall reliability to human readers; our findings demonstrate that this global assessment obscures important regional variations in performance that have direct implications that we shall take care of. The significant advantage of AI systems in deep structure assessment between +0.19 to +0.24 ICC contrasted with human superiority in insular evaluation that resulted with −0.07 ICC shows a pattern of special strengths that cannot be discerned from composite scores alone.

These region-specific findings change the direction of the proper and needed implementation strategies for AI-ASPECTS. Rather than viewing AI as a wholesale replacement for human interpretation, our findings support a hybrid reading model that integrates both strengths. For example, initial AI assessment of deep structures with targeted human verification of the insular ribbon could maximize both efficiency and accuracy, as in such a strategy, we would maintain the time-saving benefits of automated assessment while addressing the specific regions where AI performance needs to be further validated.

The technical dependencies we observed at the regional level also refine implementation guidance beyond the generalized recommendations. While the previous evidence endorsed thin-slice protocols, Overall, our findings demonstrate that this optimization primarily benefits cortical region assessment with minimal impact on deep structure evaluation [15]. This helps us to aim for more targeted protocol adjustments based on the specific brain regions of interest in individual cases and scenarios.

The primary strength of our study lies in its focused and detailed investigation and assessment of AI-driven ASPECTS performance across multiple dimensions, including software types, reference standards, time windows, and imaging factors. The previous studies have primarily reported on single software platforms in specific institutional settings, limiting generalizability [28–33]. Our results have provided a broader perspective on relative performance across different situations and settings, informing optimal application and implementation strategies for clinical practice.

Our findings extend to Nagel et al. [34], in which they reported that e-ASPECTS was non-inferior to neuroradiologists applying ASPECTS to CT scans, and Brinjikji et al. [6] study in which they reported that e-ASPECTS improved interobserver agreement among experts. By synthesizing data across multiple studies and software platforms, we provide more validated evidence that AI-driven systems match and may exceed human performance in certain conditions. The time-savings identified in our analysis (ranging from 4.6 to 11.2 minutes per case) further confirm the practical benefits of AI implementation in time-critical stroke care where rapid decision-making is essential.

Our novel approach to region-specific analysis and clinical scenario stratification represents an important advance in understanding the performance of AI-based systems. By identifying specific conditions and situations where the AI demonstrates superior performance, such as with deep brain structures and non-expert settings, we provide actionable highlights for targeted implementation that can maximize clinical benefit while acknowledging current technological limitations.

Despite our strengths demonstrated and promising findings, we have multiple limitations that shall be declared. First, significant heterogeneity was observed across studies (I^2^ values ranging from 82.7% to 93.2%), reflecting differences in study design, patient populations, reference standards, and imaging factors. While we attempted to address this point by applying the random-effects models and subgroup analyses, the heterogeneity complicates direct comparisons and may limit the generalizability.

Second, the reference standards varied across studies, including follow-up CT, MRI/DWI, and expert consensus. This variability introduces possible bias in evaluating true AI performance, as different reference standards may indicate different ground truth assessments. With a focus that MRI-based reference standards might overestimate the extent of infarction compared to initial NCCT findings, potentially affecting the real performance metrics.

Third, selection bias may be present as most included studies analyzed patients with known infarcts, which could positively affect the scoring performance for both AI and human readers. A prospective blind study, including normal brain scans, would provide a more accurate assessment of diagnostic accuracy, but such designs were not available in the included studies. Fourth, most included studies provided limited information about AI algorithm training methodologies. The risk of overfitting cannot be excluded, especially in studies that may have used the same or similar datasets for training and validation. Without transparent reporting of development and validation processes, the reliability of the AI performance to new datasets remains unclear. Finally, while our analysis of clinical scenarios provides important practical points, it was constrained by the available data from the included studies. Some conditions, such as posterior circulation involvement, had limited dedicated evidence, and our analysis relied on extrapolation from broader study findings in these areas.

Based on our findings and limitations, several key recommendations are made for future studies and applications in healthcare settings. First, we advocate for standardized reporting of AI algorithm development, including transparent descriptions of training datasets, validation methodologies, and performance metrics across individual ASPECTS regions. This standardization would facilitate more meaningful comparisons between the systems and a more reliable assessment of generalizability despite the differences between hospital and healthcare settings.

Second, future studies should focus on utilizing prospective designs with consecutive patient enrollment, including positive and negative cases, to provide more realistic assessments of AI performance in real-life practice. Stratification by important clinical variables identified in our meta-regression, such as stroke severity, would further improve the understanding of optimal implementation scenarios.

Third, our results suggest that hybrid approaches combining the strengths of AI and human interpretation may be the best possible choice, if possible. Studies aiming to investigate various collaborative models, such as AI-assisted reading with human verification of specific regions as insular ribbons, would be important to maximize accuracy while maintaining workflow efficiency.

Finally, implementation studies assessing the impact of AI-assisted ASPECTS on the outcomes and treatment decisions are needed. While our results and findings have demonstrated technical performance advantages in certain conditions, the translation of these advantages to improved patient outcomes remains to be made. Studies investigating door-to-treatment times, treatment decision accuracy, and functional outcomes with and without AI assistance would provide more reliable evidence for validation.

## 5. Conclusion

AI-based ASPECTS systems have transitioned from experimental technology to clinically viable tools that can optimize acute stroke imaging interpretation. Our findings endorse that their integration into clinical practice should be done on well-planned pathways. Three specific implementation pathways are concluded from our findings: first, as primary readers with human verification of the insular ribbon in patients with high NIHSS scores; second, as decision support tools in centers without 24/7 neuroradiology expertise; and third, as adjudicators in borderline ASPECTS cases (scores 5–7) where treatment decisions are most important.

Region-specific performance analysis demonstrated that AI systems are performing best at detecting subtle changes in deep brain structures but may miss insular ribbon involvement, which can guide targeted human verification of specific regions. Optimizing technical factors matter significantly: implementing AI with thin-slice protocols (≤2.5mm) provides higher accuracy gains than workflow adjustments alone. The projected time savings of 7–11 minutes per case for stroke centers utilizing these systems could significantly reduce the door-to-needle time while reducing reader fatigue and interpretation errors during off-hours.

Rather than viewing AI as a replacement for radiological expertise, our findings support a precision application and integrative approach where humans and AI complement each other’s strengths. AI’s consistency and deep-structure detection, paired with human expertise in interpretation, offer us a pathway to more reliable, efficient, and accurate stroke imaging assessment than either could achieve independently. As these systems continue to advance, improve, and improve over time, their targeted integration at the important decision points in the stroke care team represents a significant advancement in our ability to deliver timely and appropriate treatment to patients with acute ischemic stroke.

## Supplementary Material

Supplementary Tables

## Figures and Tables

**Table 1:** Baseline Characteristics of The Included Studies.

Study (Author, Year)	ASPECTS Software	Patients (n)	Mean/Median Age (years)	Sex (M/F)	Baseline NIHSS	Time to NCCT (min)	CT Slice Thickness (mm)	Reference Standard	Key ICC Values (AI vs Reference)
Brinjikji et al., 2021 [6]	e-ASPECTS (Brainomix)	60	67.3 ± 16.3	28/32	18 (10–22)	NR	NR	24h CT/MRI consensus	NR
Delio et al., 2021 [16]	RAPID ASPECTS (iSchemaView)	50	62.7 ± 13.2	32/18	17.5	3.4 (1.5)	2.5	MRI consensus	NR
Kuang et al., 2020 [17]	Custom-made software	100	70 (64–77)	86/71	NR	49 (23.8–95.5)	5.0	DWI within 1h	0.58 (0.35–0.61)
Hoelter et al., 2020 [8]	e-ASPECTS, RAPID, Frontier (Comparative)	131	75 (66–82)	76/55	17 (13–20)	NR	1.0	NCCT consensus	0.78 (0.65–0.87)
Wolff et al., 2020 [18]	Syngo.via Frontier (Siemens)	355	66 (54–76)	204/151	18 (15–22)	114 (68–196)	Mixed	Consensus on baseline CT	0.49 (0.41–0.57)
Neuhaus et al., 2019 [19]	e-ASPECTS (Brainomix)	178	67.6 ± 14.8	87/91	18 (12–22)	NR	5.0	NR	0.66 (0.57–0.74)
Goebel et al., 2019 [20]	Syngo.via Frontier (Siemens)	100	74.5 (30–95)	38/62	12 (2–21)	91 (32–836)	5.0	NR	0.23 (0.03–0.41)
Li et al., 2019 [21]	Syngo.via Frontier (Siemens)	55	65 (28–87)	42/13	9 (1–35)	185 (33–360)	NR	Follow-up NCCT consensus	0.65 (0.46–0.76)
Albers et al., 2019 [22]	RAPID ASPECTS (iSchemaView)	65	61 (32–79)	41/24	19 (16–23)	228 ± 114	2.5	DWI independent review	0.90 (0.75–0.86)
Guberina et al., 2018 [23]	e-ASPECTS (Brainomix)	119	70 (35–94)	NR	7 (1–21)	76 (30–120)	NR	Follow-up CT by neuroradiologist	0.69 (0.46–0.70)
Kuang et al., 2018 [24]	Custom-made software	602	71 (62–80)	309/293	15 (9–19)	114 (73–183)	5.0	24h NCCT expert measurement	0.70 (0.66–0.82)

AI: Artificial Intelligence; ASPECTS: Alberta Stroke Program Early CT Score; CT: Computed Tomography; DWI: Diffusion-Weighted Imaging; F: Female; ICC: Intraclass Correlation Coefficient; LVO: Large Vessel Occlusion; M: Male; MRI: Magnetic Resonance Imaging; NCCT: Non-Contrast Computed Tomography; NIHSS: National Institutes of Health Stroke Scale; NR: Not Reported.

**Table 2:** Subgroup Analyses of AI-driven ASPECTS Performance.

Analysis Subgroup	Total Included Patients (n)	AI vs Expert ICC (95% CI)	AI vs Reference ICC (95% CI)	Expert vs Reference ICC (95% CI)	AI Performance Advantage
Software Type: Brainomix e-ASPECTS	488	0.66 (0.57, 0.74)	0.78 (0.64, 0.87)	0.64 (0.45, 0.77)	+0.14
Software Type: RAPID ASPECTS	246	N/A	0.86 (0.78, 0.92)	0.59 (0.42, 0.73)	+0.27
Software Type: Syngo.via Frontier	642	0.50 (0.32, 0.65)	0.60 (0.48, 0.69)	0.55 (0.43, 0.65)	+0.05
Software Type: Custom software	702	0.63 (0.54, 0.71)	0.64 (0.53, 0.73)	0.66 (0.57, 0.73)	−0.02
Reference Standard: MRI/DWI-based	215	N/A	0.74 (0.57, 0.85)	0.61 (0.48, 0.72)	+0.13
Reference Standard: Follow-up CT-based	1241	N/A	0.63 (0.53, 0.71)	0.60 (0.51, 0.68)	+0.03
Reference Standard: NCCT Consensus-based	131	N/A	0.78 (0.65, 0.87)	N/A	N/A
Time Window: Early (<120 min)	219	N/A	0.64 (0.41, 0.79)	0.62 (0.44, 0.76)	+0.02
Time Window: Intermediate (120–240 min)	1022	N/A	0.70 (0.58, 0.79)	0.54 (0.40, 0.67)	+0.16
Time Window: Late (>240 min)	155	N/A	0.65 (0.38, 0.82)	0.68 (0.51, 0.80)	−0.03
CT Slice Thickness: ≤2.5mm	246	N/A	0.79 (0.69, 0.86)	0.63 (0.51, 0.73)	+0.16
CT Slice Thickness: >2.5mm	874	N/A	0.65 (0.55, 0.74)	0.56 (0.47, 0.64)	+0.09
CT Slice Thickness: Mixed/Not reported	856	N/A	0.69 (0.58, 0.77)	0.65 (0.53, 0.74)	+0.04

AI: Artificial Intelligence; ASPECTS: Alberta Stroke Program Early CT Score; CT: Computed Tomography; DWI: Diffusion-Weighted Imaging; ICC: Intraclass Correlation Coefficient; min: Minutes; MRI: Magnetic Resonance Imaging; NCCT: Non-Contrast Computed Tomography; N/A: Not Available or Not Applicable; CI: Confidence Interval.

**Table 3:** Meta-Regression Results for Predictors of AI-Reference ICC.

Variable	Coefficient	95% CI	P-value	Interpretation
NIHSS score	0.023	0.003, 0.044	0.026	Higher NIHSS associated with better AI performance
Patient age	0.008	−0.004, 0.020	0.186	Age is not significantly associated with AI performance.
Sample size	−0.0002	−0.0004, 0.0001	0.241	Sample size not significantly associated with AI performance
Publication year	0.039	−0.025, 0.103	0.232	Trend toward better performance in more recent studies

AI: Artificial Intelligence; CI: Confidence Interval; ICC: Intraclass Correlation Coefficient; NIHSS: National Institutes of Health Stroke Scale.

**Table 4:** Clinical Impact of AI-ASPECTS by Specific Scenarios.

Clinical Scenario	AI Advantage (ICC Δ)	Reader Agreement κ	Time Savings (min)	Primary Software Recommendation	Level of Evidence	Implementation Priority
High-Stakes Decision Making (ASPECTS 5–7)	+0.22	0.42	7.2	RAPID ASPECTS	Strong	Critical
Ultra-Early Assessment (<90 min)	+0.09	0.51	8.4	e-ASPECTS	Moderate	High
Late Window Evaluation (>6 hrs)	+0.18	0.47	5.9	e-ASPECTS	Moderate	High
Low NIHSS with Suspected LVO	+0.11	0.39	9.3	RAPID ASPECTS	Limited	Moderate
Non-Expert Reader Setting	+0.24	0.35	11.2	Any AI	Strong	Critical
Thin-Slice CT + High NIHSS	+0.26	0.58	6.8	RAPID ASPECTS	Strong	Critical
Wake-Up Stroke Assessment	+0.21	0.44	8.7	e-ASPECTS	Limited	Moderate
CT with Motion Artifacts	+0.19	0.38	9.5	Syngo.via Frontier	Limited	Moderate
Posterior Circulation Involvement	+0.07	0.41	4.6	Limited Data	Very Limited	Low
Prior Stroke/White Matter Disease	+0.16	0.33	7.8	e-ASPECTS	Moderate	High

AI: Artificial Intelligence; ASPECTS: Alberta Stroke Program Early CT Score; CT: Computed Tomography; ICC Δ: Intraclass Correlation Coefficient Difference; κ: Kappa Statistic (measure of inter-reader agreement); LVO: Large Vessel Occlusion; min: Minutes; NIHSS: National Institutes of Health Stroke Scale.

## Data Availability

This review article does not contain any new primary data. All information discussed is derived from previously published sources and publicly available databases, as cited in the manuscript.
