# Supplementary material for: Human Readers versus AI-Based Systems in ASPECTS Scoring for Acute Ischemic Stroke: A Systematic Review and Meta-Analysis with Region-Specific Guidance
Source: ASIDE Intern Med. Author manuscript; Available in PMC 2025 Oct 3. (PMC12490272; doi:10.71079/aside.im.05172573)
Supplement: Supplementary Tables [file NIHMS2108370-supplement-Supplementary_Tables.pdf]

Supplementary Table 1: Heterogeneity Assessment, ICC Interpretation and Publication Bias for Primary Meta-Analyses.

Analysis Type	Studies (Comparisons)	Pooled ICC (95% CI)	Heterogeneity I² (%)	Cochran's Q p-value	ICC Interpretation	Publication Bias (Egger's p-value)
Between-expert inter-rater reliability	7 (10)	0.72 (0.63, 0.79)	82.7	<0.001	Good	0.3145
Software vs expert readings	5 (7)	0.54 (0.40, 0.67)	89.3	<0.001	Moderate	0.6782
Expert readings vs reference standard	8 (12)	0.62 (0.52, 0.71)	91.0	<0.001	Good	0.6978
Software vs. reference standard	7 (9)	0.72 (0.61, 0.80)	93.2	<0.001	Good	0.2145

Notes: ICC = Interclass Correlation Coefficient; I² values >50% indicate substantial heterogeneity; ICC interpretation: poor (<0.40), moderate (0.40-0.59), good (0.60-0.74), and excellent (0.75-1.00); Egger's p-value >0.05 indicates no significant publication bias. All analyses were conducted using random effects models due to high heterogeneity.

Supplementary Table 2: QUADAS-2 Risk of Bias and Applicability Assessment.

Study (Author, Year)	*Patient Selection*	*Index Test: Automated ASPECTS*	*Reference Standard*	*Flow and Timing*	*Reference Standard (App)*	*Overall Quality Score*
Brinjikji et al., 2021	*Low*	*NR*	*Unclear*	*Low*	*Low*	*6/7 (86%)*
Delio et al., 2021	*Low*	*NR*	*Unclear*	*High*	*Low*	*5/7 (71%)*
Kuang et al., 2020	*High*	*Low*	*Unclear*	*High*	*Unclear*	*5/9 (56%)*
Hoelter et al., 2020	*Low*	*Low*	*Unclear*	*Low*	*Low*	*6/7 (86%)*
Wolff et al., 2020	*Low*	*Low*	*High*	*High*	*Low*	*7/9 (78%)*
Neuhaus et al., 2019	*Low*	*NR*	*NR*	*NR*	*NR*	*3/3 (100%)*
Goebel et al., 2019	*Low*	*NR*	*NR*	*Low*	*NR*	*4/4 (100%)*
Li et al., 2019	*Low*	*Low*	*Low*	*Low*	*Low*	*8/8 (100%)*
Albers et al., 2019	*Low*	*Low*	*Low*	*Low*	*Low*	*9/9 (100%)*
Guberina et al., 2018	*Low*	*Low*	*High*	*Low*	*Low*	*8/9 (89%)*
Kuang et al., 2018	*Unclear*	*Low*	*Unclear*	*Low*	*Low*	*5/7 (71%)*

Detailed Assessment Legend

Assessment	Description
Low Risk	The study adequately addresses this domain with minimal potential for bias.
Unclear Risk	The study provides insufficient information to determine the risk of bias in this domain.
High Risk	Study design or execution introduces potential bias in this domain.
Not Reported (NR)	The domain is not assessed or applicable in the original study.

ASPECTS: Alberta Stroke Program Early CT Score; App: Applicability; Low: Low risk of bias; High: High risk of bias; NR: Not Reported; Unclear: Unclear risk of bias or applicability concern; QUADAS-2: Quality Assessment of Diagnostic Accuracy Studies-2.
